# Genetic ancestry of participants in the National Children’s Study

**DOI:** 10.1186/gb-2014-15-2-r22

**Published:** 2014-02-03

**Authors:** Erin N Smith, Kristen Jepsen, Angelo D Arias, Peter J Shepard, Christina D Chambers, Kelly A Frazer

**Affiliations:** 1 Moores UCSD Cancer Center, University of California San Diego, 9500 Gilman Drive, La Jolla, CA 92093, USA; 2 Department of Pediatrics and Rady Children’s Hospital, University of California San Diego, 9500 Gilman Drive, La Jolla, CA 92093, USA; 3 Clinical and Translational Research Institute, University of California San Diego, 9500 Gilman Drive, La Jolla, CA 92093, USA; 4 Institute for Genomic Medicine, University of California San Diego, 9500 Gilman Drive, La Jolla, CA 92093, USA

## Abstract

**Background:**

The National Children’s Study (NCS) is a prospective epidemiological study in the USA tasked with identifying a nationally representative sample of 100,000 children, and following them from their gestation until they are 21 years of age. The objective of the study is to measure environmental and genetic influences on growth, development, and health. Determination of the ancestry of these NCS participants is important for assessing the diversity of study participants and for examining the effect of ancestry on various health outcomes.

**Results:**

We estimated the genetic ancestry of a convenience sample of 641 parents enrolled at the 7 original NCS Vanguard sites, by analyzing 30,000 markers on exome arrays, using the 1000 Genomes Project superpopulations as reference populations, and compared this with the measures of self-reported ethnicity and race. For 99% of the individuals, self-reported ethnicity and race agreed with the predicted superpopulation. NCS individuals self-reporting as Asian had genetic ancestry of either South Asian or East Asian groups, while those reporting as either Hispanic White or Hispanic Other had similar genetic ancestry. Of the 33 individuals who self-reported as Multiracial or Non-Hispanic Other, 33% matched the South Asian or East Asian groups, while these groups represented only 4.4% of the other reported categories.

**Conclusions:**

Our data suggest that self-reported ethnicity and race have some limitations in accurately capturing Hispanic and South Asian populations. Overall, however, our data indicate that despite the complexity of the US population, individuals know their ancestral origins, and that self-reported ethnicity and race is a reliable indicator of genetic ancestry.

## Background

The major goal of the National Children’s Study (NCS), authorized by the US Congress through the Child Health Act of 2000, is to discover and characterize environmental exposures that contribute to causation of disease or, conversely, that enhance children’s health (Children’s Health Act of 2000, Public Law 106–310 Sec. 1004). The pilot phase of the NCS, known as the Vanguard Study, is a small-scale study, using convenience sampling, which is being conducted to evaluate the feasibility, acceptability, and costs of the methods that will be used to carry out the main study. The Vanguard Study began in 2009 with a total of 7 locations, or Vanguard Centers, and grew to include 40 sites.

Race and ethnicity are associated with environmental risk factors for disease [1], such as tobacco smoke [2], air quality [3], and food environments [4]. While the relationship between race and genetics has been contentious [5,6], it is clear that genetic factors associated with disease can vary with racial background, resulting in common disease loci differing between ancestral groups [7]. These environmental and genetic differences could result in ethnicity and race being associated with various health outcomes, such as cancer treatment [8] and toxicology [9], and are therefore important to consider in large epidemiological studies of environmental influences on development, such as the NCS.

Efforts over the past 5 years to genotype human populations have shed light on human genetic diversity, human population evolution, and migration patterns [10-12]. In recent efforts to comprehensively identify the majority of common variation (>1%) in worldwide populations, the 1000 Genomes Project (1KG) [13] is sequencing 2,500 individuals from 25 world populations. Currently, genotype array data are available for 21 of these populations, which are classified into 5 superpopulations: African (AFR), Ad Mixed American (AMR), East Asian (ASN), European (EUR), and South Asian (SAN). Advances in genotyping technologies have yielded cost effective tools, such as the $54/sample Illumina HumanExome Array, allowing for genotyping of ancestry informative markers, the majority of genome-wide association study (GWAS)-associated loci, and rare coding variations potentially associated with disease.

Our goal was to determine whether self-reported race and ethnicity was concordant with genetic ancestry in a sample of representative American counties. Because individuals can be descended from diverse ancestries that may not be well captured in census categories or may not be similar to genetic reference groups, we aimed to identify potential systematically misclassified groups to guide downstream questionnaires and genetic assays.

To this aim, we examined whether self-reported ethnicity and race accurately assesses the genetic ancestry of participants in the NCS. DNA from 641 NCS-enrolled parents from 7 Vanguard sites was successfully assayed using exome arrays, and 29,972 markers were used for ancestry estimation. We compared the genetic profiles of the NCS participants with those of the reference populations, and determined for each individual whether self-reported race and ethnicity was consistent with their most similar 1KG superpopulation. We also examined race and ethnicity categories for which we were unable to predict a match, such as Multiracial, and used the genetic predictions to infer population groups that may not be adequately captured by the current race and ethnicity categories.

## Results and discussion

Using face-to-face interviews, self-reported race and ethnicity information was collected from 645 participants from 7 counties. Questionnaire responses allowed for two ethnicities (Hispanic or Non-Hispanic) and six race categories (Black or African American, American Indian or Alaska Native, Asian, Native Hawaiian or Other Pacific Islander, White, and Some Other Race), and multiple categories could be picked (Multiracial).

DNA from whole blood was isolated and genotyped using the Illumina HumanExome Array, with 641 samples passing quality control criteria. The HumanExome Array was designed with approximately 3,000 ancestry informative markers that distinguish between European and African American ancestry, and 1,000 markers that distinguish between European and Native American ancestry. Additional content included sites that could vary by population, but that were not chosen for ancestry informativeness, such as GWAS single nucleotide polymorphisms (SNPs), coding variation, randomly selected synonymous sites, and human leukocyte antigen (HLA) tags. To identify all sites that were informative for ancestry, we calculated informativeness [14] to distinguish between the 5 superpopulation groups of the 1KG Project and identified around 30,000 sites with positive informativeness.

For each NCS participant, we identified the most similar 1KG super population. Using the ancestry informative SNPs, we clustered the genotypes of the NCS participant with the 1KG participants using multidimensional scaling (MDS). To identify the most similar superpopulation, we created a linear discriminant model based on the top 20 dimensions of the MDS, and trained it using the 1KG data. Then, based on the model, we predicted the most likely superpopulation for each NCS participant (Table 1). We additionally performed this analysis using the 21 1KG populations for which we had data (see Additional file 1: Table S1).

**Table 1:** Superpopulation classification by self-reported ethnicity and race

**Self-report**		**Expected superpopulation**	**Most similar superpopulation**^**a,b**^				
**Ethnicity**	**Race**		**AFR**	**EUR**	**ASN**	**AMR**	**SAN**
Hispanic	African American	AFR or AMR	**5**	0	0	1	0
Non-Hispanic	African American	AFR	**29**	0	0	0	1*
Hispanic	American Indian or Alaska Native	AMR	0	0	0	**2**	0
Non-Hispanic	American Indian or Alaska Native	AMR	0	0	0	**2**	1*
Non-Hispanic	Asian	ASN or SAN	0	0	**12**	0	**9**
Non-Hispanic	Native Hawaiian or Other Pacific Islander	ASN	0	0	**4**	0	0
Hispanic	White	AMR or EUR	0	8	0	**42**	0
Non-Hispanic	White	EUR	0	**427**	0	3*	0
Hispanic	Other	AMR	1*	1*	0	**60**	0
Non-Hispanic	Other	No prediction	1	1	0	1	1
Hispanic	Multiracial	No prediction	1	1	0	2	0
Non-Hispanic	Multiracial	No prediction	3	9	7	2	3
Unknown	Multiracial	No prediction	0	0	0	1	0
		Total	40	447	23	116	15

^a^Expected groups based on self-report are indicated: African (AFR), European (EUR), East Asian (ASN), Admixed American (AMR) and South Asian (SAN).

^b^Individuals that match their reported group are indicated in bold, and individuals that did not match the reported group are indicated by an asterisk.

**Figure 1:**
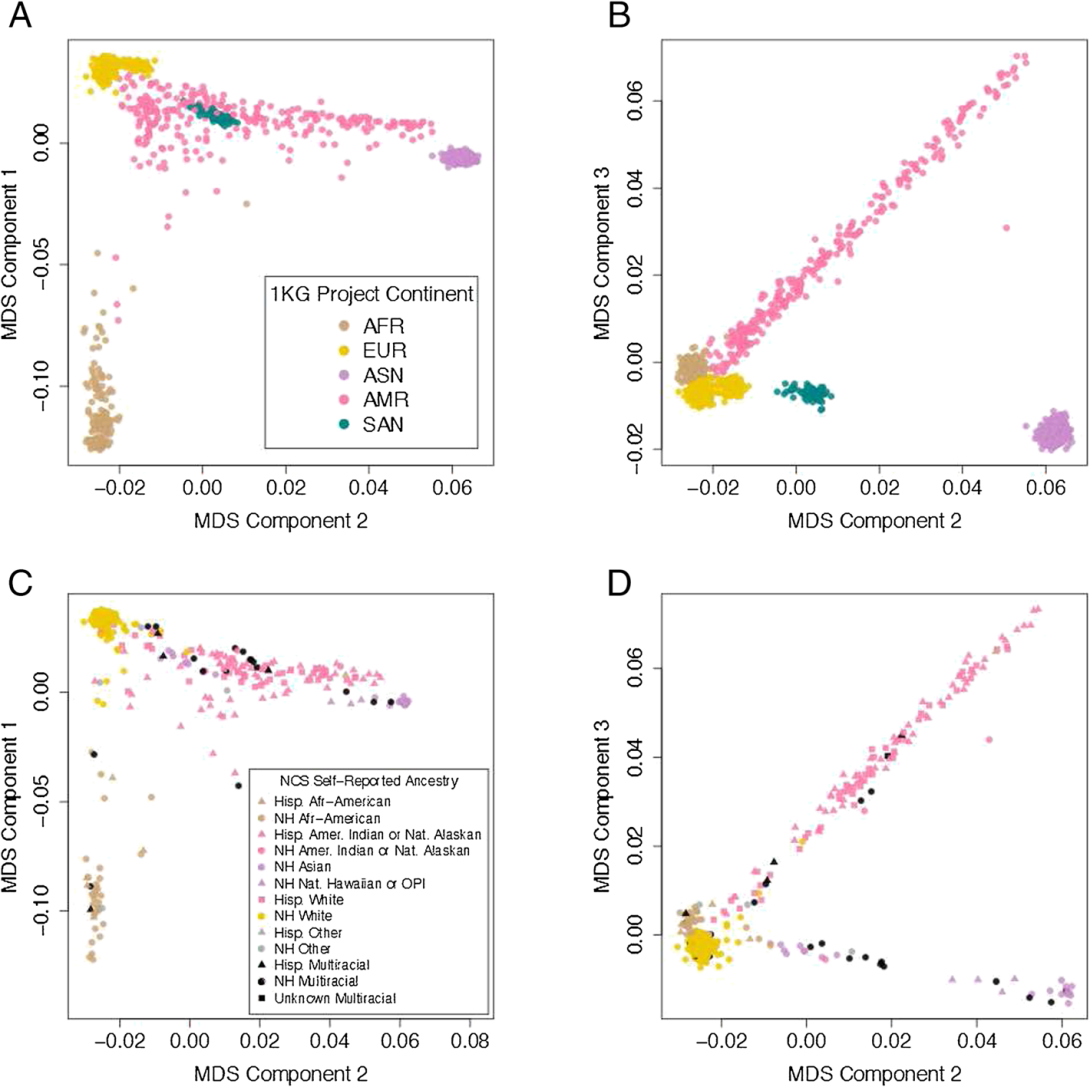
**Genetic clustering between participants in the 1000 Genomes Project (1KG) and National Children’s Study (NCS).** In total, 1445 unrelated individuals from the 1KG and 641 from the NCS were clustered on genotypic profiles using multidimensional scaling. **(A, B)** The 1KG individuals are color-coded by superpopulation and plotted according to their scores on **(A)** the first two dimensions and **(B)** the second and third dimensions. **(C,D)** NCS participants are color-coded by their expected superpopulation group and plotted according to their scores on **(C)** the first two dimensions and **(D)** the second and third dimensions. Abbreviations: Hisp, Hispanic; NH, Non-Hispanic; Afr, African; Nat. Native; Amer., American; OPI, Other Pacific Islander.

For each self-reported race and ethnic stratum, we identified which 1KG super population(s) we expected the group to match (Table 1). When multiple superpopulations were plausible, they were all included as expected matches. For example, we expected self-reported Hispanic African Americans to be most similar to either the African (AFR) or the American Admixed (AMR) 1KG superpopulations. We did not include those that identified themselves as Multiracial or Non-Hispanic Other (a total of 33 individuals) in the concordance estimates. For the NCS participants, we observed high levels of agreement between estimated genetic ancestry and self-reported ethnicity and race (Figure 1). Overall, we observed high levels of agreement between self-report and estimated ancestry, with 601/608 (98.8%) concordant calls.

Clustering can be visualized by plotting the first MDS components against each other for the 1KG (Figure 1A, B) and the NCS individuals (Figure 1C, D). Data points were plotted in the first and second dimensions (Figure 1A,C) and in the second and third dimensions (Figure 1B,D). The results showed that AFR, EUR, and ASN superpopulations are clearly differentiated in the first two dimensions, while the SAN and AMR groups are overlapping, reflecting their historical European and East Asian ancestry (Figure 1A). While the AMR group is broadly distributed, indicating that some individuals are genetically more similar to the EUR group and others to either the ASN or AFR groups, individuals in the SAN group cluster together. In the second and third dimensions, SAN and AMR are distinctly identifiable (Figure 1B). NCS individuals identified as Asian by self-report overlap with both the SAN and ASN groups. This is expected, as a distinct racial category for persons of South Asian descent (largely Indian) was not available as a self-reported race category.

We further investigated individuals that were discordant with our predictions. Linear discriminant analysis provides a relative score for how well each individual matches each group, and we observed that discordant individuals often matched their second-best superpopulation prediction. Of the seven discordantly assigned individuals, six matched their second most likely super population group, and the remaining one matched their third most likely group. We also examined our analysis of the 21 1KG populations (see Additional file 1: Table S1), and observed that 4 of the 7 discordant individuals matched a population that was in their best-matched superpopulation by self-report, even though they were not placed in that group when the 5 1KG superpopulations were used for the analysis. This suggests that in some cases, analyses at population level may be more accurate for assigning genetic ancestry to an individual than analyses at superpopulation level. Overall, however, we observed the same level of concordance using populations as we did using superpopulations (601/608, 98.8%). Discordant individuals were not likely to be the result of misidentified samples, because these individuals were collected from five of the seven NCS sites, and were not consistent with swaps within each site (data not shown).

Hispanic White and Hispanic Other self-reported groups were determined to be of closely related ancestry, with 78% of Hispanic White and 94% of Hispanic Other predicted to match the AMR population. However, individuals with a self-report of Hispanic White were more likely to match the EUR group (22%) than the Hispanic Other (6%), which is consistent with individuals that identify as Hispanic having a heritage that includes European and often, but not always, Native American ancestry.

While there was no expected population group for the 33 individuals who reported being Multiracial or Non-Hispanic Other, we were able to assign them to their most similar superpopulations. As a group, they showed great diversity, with individuals matching to each of the five superpopulations. Of note, 11 (33%) of these individuals matched to the ASN or SAN groups, which were less represented in the other categories (27/609, 4.4%). These data suggest that individuals of South Asian or East Asian descent may not adequately be captured by the NCS ethnicity and race categories.

Comparison of reported ethnicity and race with genetic ancestry highlighted the difficulties in properly capturing this information for individuals from populations with historical admixture. For the Non-Hispanic Asian population, we observed two clearly distinct populations: those closely related to the ASN population, which is composed of Han Chinese individuals (from Beijing and Southern China), Chinese individuals from Denver (CO), Japanese individuals, and Kinh individuals from Ho Chi Minh City (Vietnam); and those closely related to the SAN population, which is a population composed of Gujarati Indian individuals from Texas (Figure 1; see Additional file 1). While of related ancestry, these two populations can be clearly discriminated genetically, and the currently used race category of ‘Asian’ does not adequately distinguish between individuals of South Asian versus East Asian descent, highlighting the relevance of using genetically determined ancestry rather than self-reported ancestry alone.

A comparison of genetic ancestry to self-reported ethnicity and race for Hispanic individuals determined that the genetic ancestry of those choosing the categories of Hispanic, White (50 persons) and Hispanic, Other (62 persons) is largely the same. Individuals choosing Hispanic, White or Hispanic, Other were most similar to the AMR superpopulation (102/112) (composed of Colombian individuals in Medellin, Colombia; Mexican individuals from Los Angeles, CA; Peruvian individuals in Lima, Peru; and Puerto Rican individuals in Puerto Rico) [13], with the remaining individuals matching the European or African superpopulations.

## Conclusions

In summary, we have successfully used the Illumina HumanExome Array to classify NCS participants accurately into superpopulation ancestry groups, consistent with self-report. Refinements to self-reported ethnicity and race options for both the Non-Hispanic Asian and the Hispanic White/Hispanic Other populations would result in more accurate determination of the genetic ancestry of these populations.

## Materials and methods

### Study population

In total, 646 blood samples from parents enrolled in the NCS were obtained from the NCS biorepository. The samples were collected from seven different NCS Vanguard Centers across the USA, including Brookings County, SD (which also enrolled participants from Yellow Medicine County, MN; Pipestone County, MN; and Lincoln County, MN), Duplin County, NC; Montgomery County, PA; Orange County, CA; Queens, NY; Salt Lake County, UT; and Waukesha County, WI. A total of 710 mothers and 451 fathers were enrolled, from which 346 mothers and 300 fathers were selected for the current study. Ethnicity/race was self-described. Participants were asked during a face-to-face interview about their ethnicity and race, choosing between two ethnicity categories (Hispanic or Non-Hispanic), and six race categories (Black or African American, American Indian or Alaskan Native, Asian, Native Hawaiian or Other Pacific Islander, White, or Some Other Race), from which multiple categories could be picked (Multiracial). All participants had provided written informed consent for the use of these samples and the study was approved through the local site and/or the NCS federated institutional review boards. It should be noted that that not all NCS participants consented to providing biological samples, so the overall diversity of enrollment in the NCS may differ slightly from what we report here.

Of the 646 samples, 346 were from mothers and 300 were from fathers; 360 individuals were paired participants (mother and father), and the remaining individuals were a single enrolled parent (166 mothers, 120 fathers). Samples consisted of 200 μl of EDTA-treated whole blood for mothers, and 2.0 ml of acid citrate dextrose (ACD)-treated whole blood diluted with 2.0 ml 20% DMSO in RPMI medium (4.0 ml total volume) for fathers. Although blood sample storage methods varied between enrolled mothers and fathers, both were sufficient for the studies described here.

### DNA isolation

DNA was isolated from 200 ul of provided whole blood sample using a QIAcube and the QIAamp DNA Blood Mini QIAcube Kit (Qiagen, Valencia, CA, USA). The standard QIAcube isolation program was used, except that the elution volume was modified from 100 μl to 25 μl.

DNA concentrations ranged from 0.81 to 292 ng/μl for samples from mothers, and from 2.4 to 97.7 ng/μl for samples from fathers. For mothers, a mean (± SD) DNA concentration of 68.2 ± 51.4 ng/μl was obtained. For fathers, the mean DNA concentration was 30.5 ± 18.3 ng/μL. There was one sample (mother) from which no DNA could be isolated, resulting in 645 of 646 samples with successful isolation of DNA.

### Illumina infinium HD HumanExome BeadChip assay

Using the Illumina Infinium HD HumanExome BeadChip Assay, 6 μl each DNA sample was analyzed. Samples were processed according the manufacturer’s specifications. We observed an average SNP call rate of 99.2% per sample. Three samples (all mothers) had SNP call rates below the 90% and therefore failed quality control. One individual was subsequently removed due to high genome-wide similarity (proportion identical by descent (PI_HAT) approximately 1) to another sample. Overall, we successfully screened 641 of the 645 DNA samples (failure rate of <0.5%).

The HumanExome BeadChip was designed through a collaborative effort of multiple academic groups ([15]) in order to capture rare and common coding variation. It includes over 240,000 variants identified from diverse populations, and in addition to coding variants, includes ancestry informative markers (n = 3,468), SNPs associated with a range of common conditions, such as type 2 diabetes, cancer, metabolic, and psychiatric disorders (n = 4,761 SNPs), and additional sites of scientific interest. Because the array was designed to capture coding variants, it captures only 10% of common variation through linkage disequilibrium (r^2^ > 0.8).

### HumanExome array processing

Genotypes were called using GenomeStudio (v2011.1). Briefly, genotype intensities were reclustered together across all samples, and default criteria for genotype quality (GenCall Score >0.15) was used to filter poorly called genotypes. Genotypes were converted from Illumina TOP orientation to genome orientation (b37) using the HumanExome-12v1_A files generated through the Wellcome Trust Center for Human Genetics ([16]). Sites reported as ‘Cautious Sites’ ([17]) were removed. Sites were annotated to dbSNP 135 identifiers using The Genome Analysis Toolkit (GATK) [18].

### Data quality control

Data generated on the Illumina HiScan system was subjected to three basic quality control measures. Initially, a qualitative assessment of the assay performance was determined by visual inspection of the internal control probes on the array to ensure effective staining, hybridization, base extension, and washing. Samples were required to have a call rate of 90% or greater (three samples failed). Finally, to identify potential sample misidentification, the reported sex was compared with the sex calculated from homozygosity estimates across all SNPs on the X chromosome with a MAF >0.1 (no samples were assigned the wrong sex).

### Ancestry estimation

#### Reference populations

We used the participants of the 1KG [13] as a reference population for ancestry identification. These individuals derive from 21 different population groups covering five superpopulations: African (AFR), East Asian (ASN), South Asian (SAN), European (EUR), and Ad Mixed American (AMR) (see Additional file 1). We obtained genetic data for 1,445 unrelated individuals profiled on the Illumina Omni 2.5 array ([19]), and annotated sites to dbSNP 135 identifiers using GATK [18]. We focused on the 41,572 sites that overlapped those on the HumanExome Array.

#### Ancestry informative markers

We prioritized markers by their ability to distinguish ancestry and for being independent of each other. We calculated informativeness [14] based on the five 1KG superpopulations (AFR, ASN, SAN, EUR, and AMR) and chose markers in order of informativeness that were in linkage equilibrium (r^2^ < 0.2) with previously chosen markers within 1 Mb. Sites were not filtered based on a minor allele threshold. We ultimately identified 29, 972 markers that were shared between the Omni 2.5 array and the HumanExome array for use in ancestry estimation.

To estimate ancestry, we identified for each NCS participant the most similar 1KG superpopulation group and population group (Table 1; see Additional file 1). Specifically, we clustered genotypes from all participants at ancestry informative markers using MDS in PLINK [20]. We then developed a model based on the first 20 MDS components using linear discriminant analysis (lda command in MASS package [21] in R) with the 1KG individuals as a training set. We next used the NCS individuals as a test dataset and predicted the most likely superpopulation and population groups for each participant. We compared predicted groups with groups based on self-reported ethnicity and race. Because there was not a 1:1 relationship between self-reported ethnicity and race and the 1KG superpopulation groups, we developed expected relationships (Table 1). Individuals were considered discordant if their groups disagreed with the expected superpopulation assignments.

## Competing interests

The authors declare that they have no competing interests.

## Authors’ contributions

ENS performed the data analysis and wrote the manuscript. KJ performed the experimental assays and wrote the manuscript. ADA performed the experimental assays. PJS performed quality control analyses. CDC coordinated sample selection. KAF conceived of the study design and wrote the manuscript. All authors read and approved the final manuscript.

## Supplementary Material

Additional file 1 **Table listing the expected groups based on self-report and the population each individual matches.** Genotype and phenotype data are available through dbGaP: http://www.ncbi.nlm.nih.gov/projects/gap/cgi-bin/study.cgi?study_id=phs000662.v1.p1. Click here for file
